# Bromocriptine-QR Therapy Reduces Sympathetic Tone and Ameliorates a Pro-Oxidative/Pro-Inflammatory Phenotype in Peripheral Blood Mononuclear Cells and Plasma of Type 2 Diabetes Subjects

**DOI:** 10.3390/ijms23168851

**Published:** 2022-08-09

**Authors:** Anthony H. Cincotta, Eugenio Cersosimo, Mariam Alatrach, Michael Ezrokhi, Christina Agyin, John Adams, Robert Chilton, Curtis Triplitt, Bindu Chamarthi, Nicholas Cominos, Ralph A. DeFronzo

**Affiliations:** 1 VeroScience LLC, Tiverton, RI 02878, USA; 2 Texas Diabetes Institute, University Health System, San Antonio, TX 78207, USA; 3 Division of Diabetes, Department of Medicine, University of Texas Health Science Center at San Antonio, San Antonio, TX 78229, USA

**Keywords:** bromocriptine, vascular protection, oxidative stress, sterile inflammation, cardiovascular disease risk, type 2 diabetes, sympathetic, dopamine

## Abstract

Bromocriptine-QR is a sympatholytic dopamine D2 agonist for the treatment of type 2 diabetes that has demonstrated rapid (within 1 year) substantial reductions in adverse cardiovascular events in this population by as yet incompletely delineated mechanisms. However, a chronic state of elevated sympathetic nervous system activity and central hypodopaminergic function has been demonstrated to potentiate an immune system pro-oxidative/pro-inflammatory condition and this immune phenotype is known to contribute significantly to the advancement of cardiovascular disease (CVD). Therefore, the possibility exists that bromocriptine-QR therapy may reduce adverse cardiovascular events in type 2 diabetes subjects via attenuation of this underlying chronic pro-oxidative/pro-inflammatory state. The present study was undertaken to assess the impact of bromocriptine-QR on a wide range of immune pro-oxidative/pro-inflammatory biochemical pathways and genes known to be operative in the genesis and progression of CVD. Inflammatory peripheral blood mononuclear cell biology is both a significant contributor to cardiovascular disease and also a marker of the body’s systemic pro-inflammatory status. Therefore, this study investigated the effects of 4-month circadian-timed (within 2 h of waking in the morning) bromocriptine-QR therapy (3.2 mg/day) in type 2 diabetes subjects whose glycemia was not optimally controlled on the glucagon-like peptide 1 receptor agonist on (i) gene expression status (via qPCR) of a wide array of mononuclear cell pro-oxidative/pro-inflammatory genes known to participate in the genesis and progression of CVD (*OXR1*, *NRF2*, *NQO1*, *SOD1*, *SOD2*, *CAT*, *GSR*, *GPX1*, *GPX4*, *GCH1*, *HMOX1*, *BiP*, *EIF2α*, *ATF4*, *PERK*, *XBP1*, *ATF6*, *CHOP*, *GSK3β*, *NFkB*, *TXNIP*, *PIN1*, *BECN1*, *TLR2*, *TLR4*, *TLR10*, *MAPK8*, *NLRP3*, *CCR2*, *GCR*, *L-selectin*, *VCAM1*, *ICAM1*) and (ii) humoral measures of sympathetic tone (norepinephrine and normetanephrine), whole-body oxidative stress (nitrotyrosine, TBARS), and pro-inflammatory factors (IL-1β, IL-6, IL-18, MCP-1, prolactin, C-reactive protein [CRP]). Relative to pre-treatment status, 4 months of bromocriptine-QR therapy resulted in significant reductions of mRNA levels in PBMC endoplasmic reticulum stress-unfolded protein response effectors [*GRP78/BiP* (34%), *EIF2α* (32%), *ATF4* (29%), *XBP1* (25%), *PIN1* (14%), *BECN1* (23%)], oxidative stress response proteins [*OXR1* (31%), *NRF2* (32%), *NQO1* (39%), *SOD1* (52%), *CAT* (26%), *GPX1* (33%), *GPX4* (31%), *GCH1* (30%), *HMOX1* (40%)], mRNA levels of TLR pro-inflammatory pathway proteins [*TLR2* (46%), *TLR4* (20%), *GSK3β* (19%), *NFkB* (33%), *TXNIP* (18%), *NLRP3* (32%), *CCR2* (24%), *GCR* (28%)], mRNA levels of pro-inflammatory cellular receptor proteins *CCR2* and *GCR* by 24% and 28%, and adhesion molecule proteins *L-selectin* (35%) and *VCAM1* (24%). Relative to baseline, bromocriptine-QR therapy also significantly reduced plasma levels of norepinephrine and normetanephrine by 33% and 22%, respectively, plasma pro-oxidative markers nitrotyrosine and TBARS by 13% and 10%, respectively, and pro-inflammatory factors IL-18, MCP1, IL-1β, prolactin, and CRP by 21%,13%, 12%, 42%, and 45%, respectively. These findings suggest a unique role for circadian-timed bromocriptine-QR sympatholytic dopamine agonist therapy in reducing systemic low-grade sterile inflammation to thereby reduce cardiovascular disease risk.

## 1. Introduction

Bromocriptine-QR is a unique formulation of micronized bromocriptine mesylate, a potent sympatholytic, dopamine D2 receptor agonist that is approved for the treatment of type 2 diabetes (T2D) by the U.S. Food and Drug Administration [1,2]. In a large, randomized trial of T2D subjects, the majority (75%) of whom were without pre-existing cardiovascular disease (CVD), the circadian-timed (within 2 h of morning waking) administration of this therapy significantly reduced cardiovascular events by 40–55% over a single year [3,4,5,6]. Because these protective cardiovascular (CV) effects could not be explained by major changes in plasma lipids, HbA1c, or blood pressure, a plausible explanation for the beneficial CV effect may be related to the reduced chronic overactivation of sympathetic tone [7,8,9,10,11], a pathophysiologic abnormality that both causes and exacerbates the metabolic syndrome (MS), T2D and CVD [12,13,14,15,16,17,18]. Although poorly appreciated, the scientific literature is replete with studies that corroborate a prominent role of the **chronic** elevation of sympathetic nervous system (SNS) tone in the genesis and amplification of cellular/tissue oxidative stress and the resultant inflammation, particularly within the immune system [19,20,21,22,23,24,25,26,27,28,29,30,31,32,33,34,35,36], which promotes insulin resistance and CVD [14,30,31,32,33,34,37,38,39,40,41,42,43,44,45,46,47,48,49]. Likewise, low central dopaminergic activity, particularly at the D2 receptor, is associated with a pro-oxidative/pro-inflammatory (PO/PI) state in multiple tissues including the immune system [50,51,52,53,54,55,56,57,58,59,60,61,62,63,64,65,66]. This hypo-dopaminergic PO/PI state can result from a loss of dopaminergic inhibition of central sympathetic outflow, as well as from a direct reduction of D2 receptor activation in specific brain tissues and peripheral sites. Conversely, bromocriptine has been shown to elicit anti-inflammatory responses in the treatment of lupus, rheumatoid arthritis, uveitis, and postpartum cardiomyopathy [67,68,69,70,71,72,73,74,75], suggesting that its sympatholytic, dopaminergic actions might promote anti-inflammatory effects in T2D. Multiple studies have highlighted the critical role of the daily circadian rhythm of the central nervous system (CNS)’s dopaminergic activity in the maintenance of metabolic health (reviewed in ref [1]). Therefore, the present investigation was undertaken to assess the impact of circadian-timed bromocriptine-QR on the indicators of sympathetic tone, gene expression in peripheral blood mononuclear cells (PBMC), and plasma factors that are indicative of a PO/PI state in T2D subjects. We hypothesized, based on the basic scientific and clinical observations described above, that bromocriptine-QR treatment would reduce plasma norepinephrine/normetanephrine levels and reduce plasma and peripheral blood mononuclear cells’ (PBMC) biochemical elements involved in the generation of a pro-oxidative/pro-inflammatory state, explaining at least in part, the observed cardioprotective effect of the drug [3,4,5,6].

Although increased SNS and decreased dopaminergic tone can induce an immune and metabolic tissue PO/PI state that potentiates MS and CVD, it is also true that MS components (including hyperglycemia, insulin resistance, dyslipidemia, elevated plasma FFA levels, increased renin–angiotensin system activity, hypertension, elevated plasma thrombin, endothelin 1, and angiotensin 2 levels, and obesity) feedback to potentiate the PO/PI state within both immune and non-immune cell types. This reverberating circuit in turn can contribute to the onset and exacerbation of MS, T2D, and CVD [14,37,38,40,41,42,44,46,47,48,49,76,77,78,79,80,81,82,83,84,85,86]. Thus, a systemic **chronic** increase in the immune PO/PI state (initiated via an autonomic imbalance of elevated SNS tone and/or metabolic influences) fuels a whole-body shift in cardiometabolic status from health toward MS, T2D, and CVD, setting up a self-sustaining cycle of whole-body cardiometabolic pathology [84,87,88,89,90,91,92,93,94,95,96,97,98,99,100]. Studies of MS subjects with and without T2D have provided evidence that the PO/PI state of PBMCs contributes to the development of MS and CVD [77,78,79,80,81,84,86,101,102,103]. Two major cellular dysfunctions that interact to contribute to an immunocyte (e.g., PBMCs) PO/PI state are endoplasmic reticulum (ER) stress and toll-like receptor (TLR) pro-inflammatory pathway overactivation.

In a recent study, we demonstrated that bromocriptine-QR treatment in inadequately controlled T2D subjects treated with a glucagon-like peptide 1 receptor agonist (GLP-1RA) improved endothelial dysfunction, reduced blood pressure, and enhanced suppression of post-meal hepatic glucose production without increasing plasma insulin-effects suggestive of reduced SNS tone [104]. The present investigation was undertaken with this same study population to assess the impact of this 4-month treatment with bromocriptine-QR on PBMC PO/PI status by evaluating the mRNA expression of a series of genes involved in or counter-responsive to a PO/PI state. These genes include those coding for endoplasmic reticulum (ER) stress response proteins, proteins responsive to a pro-oxidative environment (i.e., antioxidant gene expression), TLR cascade activity proteins, pro-inflammatory protein inducers, and pro-inflammatory adhesion molecules. Additionally, we investigated the impact of bromocriptine-QR on the plasma biomarkers of systemic pro-oxidative stress (thiobarbituric acid reactive substances (TBARS), nitrotyrosine), pro-inflammatory factors (IL-18, IL-6, MCP1, IL-1β, prolactin, CRP) and sympathetic tone (norepinephrine, normetanephrine) to complement these PBMC gene expression studies in order to gain a more complete picture of the whole-body PO/PI mechanisms that could explain the CVD protective effect of this anti-diabetes therapy. A more detailed explanation of the rationale of the specific PBMC mRNAs of the ER stress and TLR activation pathways and plasma factors examined in this study are provided in the Methods section.

## 2. Results

### 2.1. Baseline Characteristics of Study Subjects

The baseline demographic and metabolic characteristics of the study subjects are presented in Table 1.

The study utilized open recruitment for the T2D subjects but due to the high Hispanic population in the vicinity of the study site (San Antonio, TX, USA) and the high rate of T2D among Hispanics, the enrollment population resulted in being all Hispanic.

### 2.2. Bromocriptine-QR Effect on Plasma Levels of SNS Tone Markers, Pro-Oxidative Stress Markers, and Pro-Inflammatory Factors

**Figure 1:**
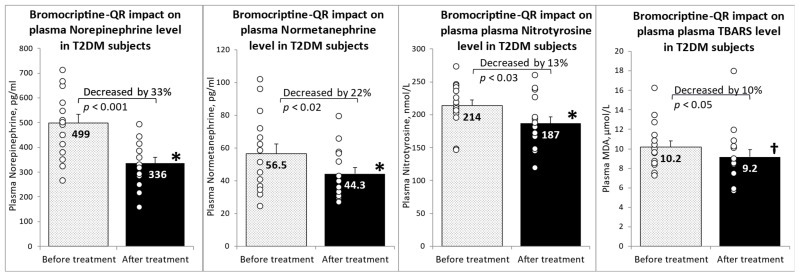
Effect (change from baseline) of 4 months of bromocriptine-QR treatment on plasma markers of elevated sympathetic tone and oxidative stress. * Significant, 2-tailed *t*-test; † Significant, 1-tailed *t*-test.

Following 4 months of timed (within 2 h of waking in the morning) daily bromocriptine-QR therapy, the fasting plasma levels of norepinephrine decreased by 33% (from 499 to 336 pg/mL, *p* < 0.001), and those of normetanephrine decreased by 22% (from 56.5 to 44.3 pg/mL, *p* < 0.02). Such treatment reduced the plasma systemic oxidative stress marker nitrotyrosine by 13% (from 214 to 187 nmol/L, *p* < 0.03) and its related downstream oxidative stress marker TBARS by 10% (from 10.2 to 9.2 μM/L MDA, *p* < 0.05 1-tailed) (Figure 1).

**Figure 2:**
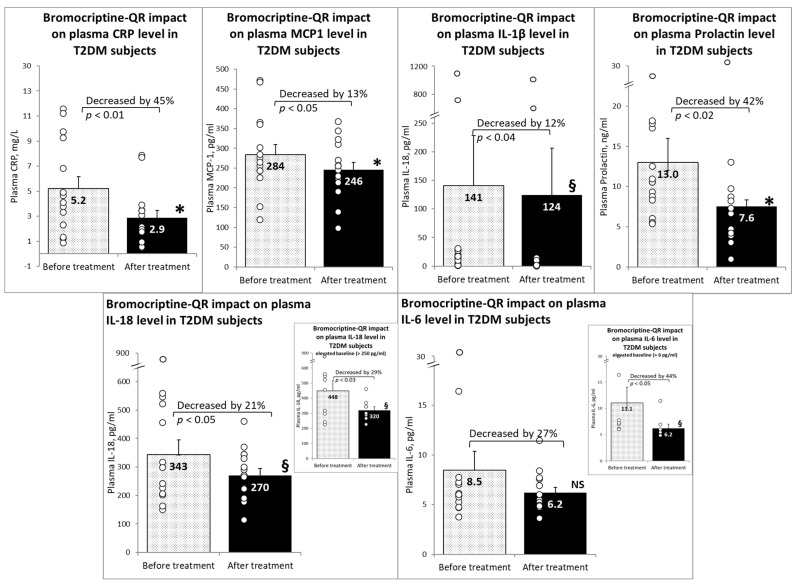
Effect (change from baseline) of 4 months of bromocriptine-QR treatment on plasma inflammatory markers. * Significant, 2-tailed *t*-test; § Significant, Wilcoxon signed rank test; NS, Not Significant.

Furthermore, bromocriptine-QR therapy reduced the plasma levels of the pro-inflammatory factors MCP1 (by 13% from 284 to 246 pg/mL, *p* < 0.05), IL-1β (by 12% from 141 to 124 pg/mL, *p* < 0.04), prolactin (by 42% from 13.0 to 4.6 ng/mL, *p* < 0.02), IL-18 (by 21% from 343 to 270 pg/mL, *p* < 0.03), and CRP (by 45% from 5.2 to 2.9 mg/L). There was a non-significant trend toward a reduction in plasma IL-6 levels following bromocriptine-QR treatment (by 27% from 8.5 to 6.2 pg/mL, NS). However, among subjects with an elevated baseline lL-6 level (>6 pg/mL), the plasma IL-6 level decreased by 44% (from 11.1 to 6.2 pg/mL, *p* < 0.02) (Figure 2).

### 2.3. Effects of Bromocriptine-QR Therapy on mRNA Expression of PBMC ER Stress-Associated Unfolded Protein Response Genes

**Figure 3:**
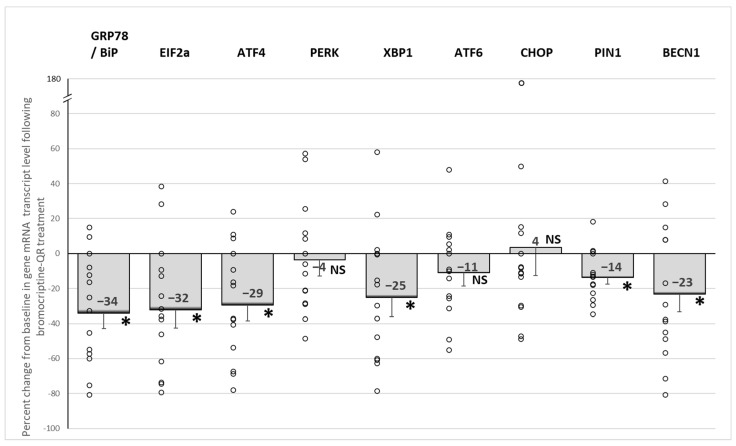
Effect (change from baseline) of 4 months of bromocriptine-QR treatment on PBMC ER stress-associated unfolded protein response gene mRNA level. * Significant, 2-tailed *t*-test; NS, Not Significant.

Bromocriptine-QR therapy reduced the expression of *GRP78/BiP*, which acts as the initiation protein for the ER-unfolded protein response via the release and thus activation of its tethered stress sensor proteins *PERK*, IER1, and ATF6 by 34% (*p* < 0.002), as well as the gene expression levels of the major downstream signal transduction amplifiers of the ER stress response responsible for maintaining cell viability: *EIF2α*, *ATF4*, and *XBP1* by 32% (*p* < 0.01), 29% (*p* < 0.01), and 25% (*p* < 0.04), respectively. Similarly, the ER stress response survival protein *BECN1*, responsible for ER stress-induced autophagy activation, and *PIN1*, responsible for DNA repair and inflammation activation, were reduced by bromocriptine-QR by 23% (*p* < 0.04) and 14% (*p* < 0.005), respectively. However, such treatment was without an effect on the levels of the GRP78-tethered activators *PERK* (4% decrease, NS) or *ATF6* (11% decrease, NS) or their downstream target transcription factors *MAPK8/JNK* (1% decrease, NS) and *CHOP* (+4%, NS)- pathways largely responsible for the initiation of cellular apoptosis (Figure 3).

### 2.4. Effect of Bromocriptine-QR Therapy on mRNA Expression of PBMC Oxidative Stress Response (Anti-Oxidant) Genes

**Figure 4:**
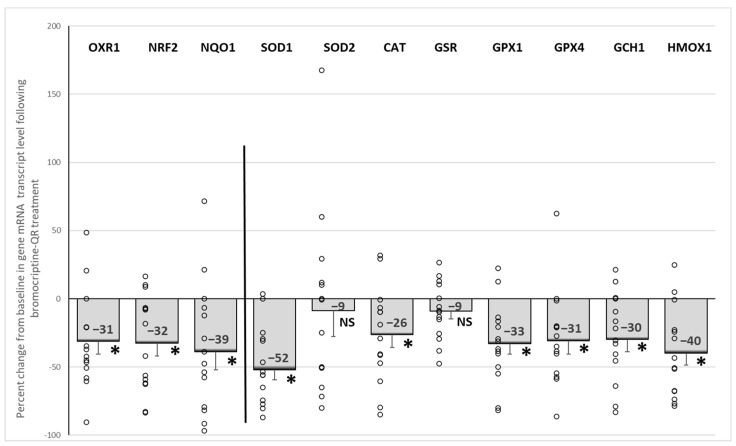
Effect (change from baseline) of 4 months of bromocriptine-QR treatment on the PBMC oxidative stress response (antioxidant) gene mRNA level. * Significant, 2-tailed *t*-test; NS, Not Significant.

Bromocriptine-QR therapy reduced the mRNA expression of the master oxidative stress response (antioxidant) gene regulators, *NRF2, OXR1*, and *NQO1* by 32% (*p* < 0.005), 31% (*p* < 0.006), and 39% (*p* < 0.02), respectively. Likewise, such treatment also reduced the target oxidative stress response genes of these master antioxidant gene regulators, including *SOD1* (by 52%, *p* < 0.0001), *CAT* (by 26%, *p* < 0.02), *GPX1* (by 33%, *p* < 0.01), and *GPX4* (by 31%, *p* < 0.01), without an effect on either *SOD2* (9% decrease, NS) or *GSR* (9% decrease, NS). Consistent with this drug-induced reduction in the oxidative stress response gene expression, the expression of the two major oxidative stress response genes, *GCH1* (by 30%, *p* < 0.01) and *HMOX1*, were also reduced (by 40%, *p* < 0.001) (Figure 4).

### 2.5. Effect of Bromocriptine-QR Therapy on mRNA Expression of PBMC Pro-Inflammatory Genes

**Figure 5:**
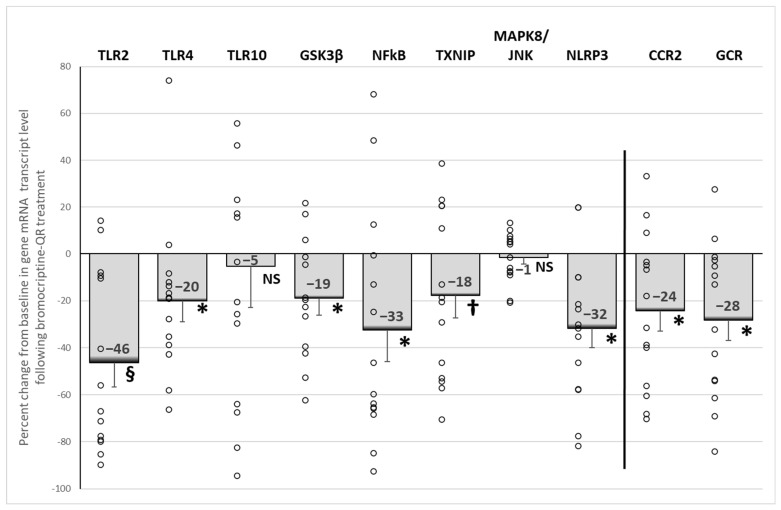
Effect (change from baseline) of 4 months of bromocriptine-QR treatment on PBMC Proinflammatory gene mRNA level. * Significant, 2-tailed *t*-test; † Significant, 1-tailed *t*-test; § Significant, Wilcoxon signed rank test; NS, Not Significant.

Bromocriptine-QR therapy reduced the mRNA expression of the damage-associated molecular pattern (DAMP)-activated pro-inflammatory pathway initiators *TLR2* (by 46%, *p* < 0.001) and *TLR4* (by 20%, *p* < 0.05), without an effect on *TLR10* (5% decrease, NS). The treatment also reduced the mRNA expression of the TLR downstream pro-inflammatory gene signaling pathway proteins *GSK3β* (by 19%, *p* < 0.03), *NFkB* (by 33%, *p* < 0.03), *TXNIP* (by 18%, *p* < 0.05, 1-tailed), and *NLRP3* (by 32%, *p* < 0.003), as well as the expression of the PBMC pro-inflammatory initiation receptors, *CCR2* (by 24%, *p* < 0.02) and *GCR* (by 28%, *p* < 0.01) (Figure 5).

### 2.6. Effect of Bromocriptine-QR Therapy on mRNA Expression of PBMC Secretory Adhesion Molecule Genes

**Figure 6:**
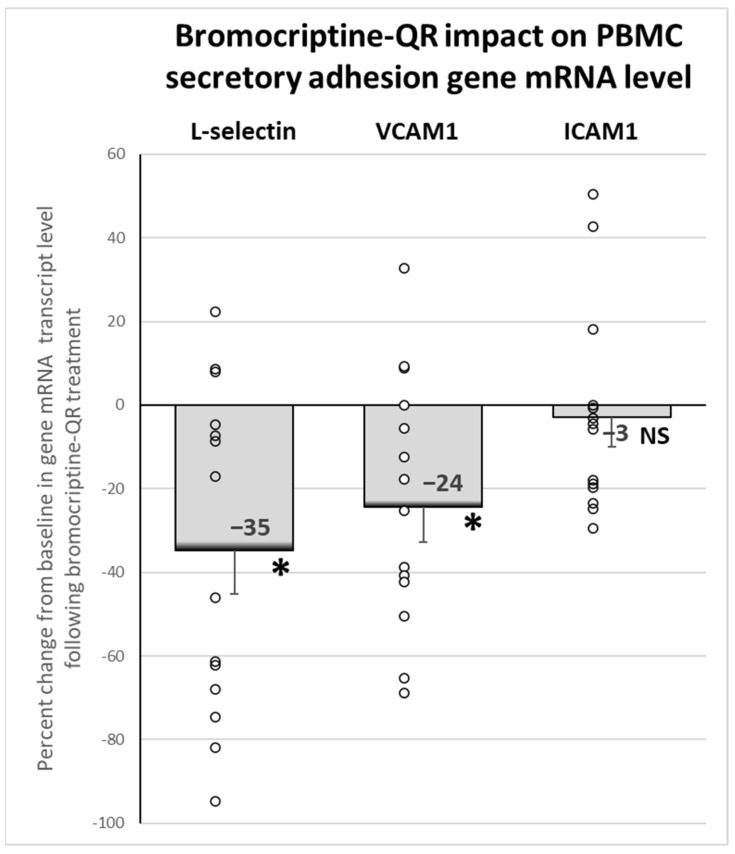
Effect (change from baseline) of 4 months of bromocriptine-QR treatment on PBMC Secretory Adhesion Molecule gene mRNA level. * Significant, 2-tailed *t*-test; NS, Not Significant.

Bromocriptine-QR therapy reduced the mRNA expression of the secretory adhesion molecule genes: L-selectin (*SELL,* by 35%, *p* < 0.02) and vascular cell adhesion molecule 1 (*vCAM,* by 24%, *p* < 0.02), without an effect on intercellular adhesion molecule 1 (*iCAM,* 3% decrease, NS), Figure 6.

There was a significant positive correlation between the change in the plasma total measured catecholamines (norepinephrine plus normetanephrine) level and the change in the PBMC *NLRP3* expression (Pearson’s *r* = 0.75, *p* = 0.002) and the change in the *EIF2α* expression (Pearson’s *r* = 0.56, *p* = 0.047) from baseline to week 24 of bromocriptine-QR therapy. However, there was no significant such correlation between the change in the plasma norepinephrine plus normetanephrine level and the PBMC *NRF2* expression. There was a negative correlation between the change from baseline in fasting RHI and the change in HbA1c (Kendall’s tau-b, *τ*_b_ = −0.42) (i.e., reduction in HbA1c correlates with a reduction in endothelial dysfunction), which was statistically significant (*p* = 0.041).

### 2.7. Adverse Effects

The adverse effects were as previously described in [104]. Cycloset was generally well tolerated. The Cycloset dose was decreased to 2.4 mg/day for symptoms of either orthostatic hypotension, lightheadedness, nausea, or vomiting in three patients (20% of the study population).

## 3. Discussion

This study is the first to demonstrate that bromocriptine-QR treatment of T2D subjects is associated with a widespread reduction in the expression of PBMC genes associated with an active systemic pro-oxidative/pro-inflammatory state. Bromocriptine-QR treatment for 4 months caused a simultaneous reduction in the ER stress genes, oxidative stress response genes, and TLR pathway proinflammatory genes, concurrent with a reduction in the plasma biomarkers of oxidative stress and inflammation and sympatholytic effects to reduce plasma norepinephrine and normetanephrine levels in T2D subjects. The effect was observed in a mixed-study population of T2D males and females (including post-menopausal females). To our knowledge, this is the first demonstration of these cumulative effects in response to any anti-diabetes agent.

Bromocriptine-QR therapy reduced the gene expression of several PBMC PO/PI signature genes including the ER stress-response proteins *GRP78*, *XBP1*, *EIF2α*, and *ATF4* that function as part of the unfolded protein response (UPR) to alleviate ER (oxidative) stress [89,102,105,106,107]. However, such treatment did not influence the *PERK*→*CHOP*, *ATF6*→*CHOP*, or ER stress→*JNK* gene expression levels that couple to apoptosis [105], suggesting the presence of a chronic low-grade, pro-inflammatory, cellular/ER stress response that was attenuated by the bromocriptine-QR therapy. It should also be noted that sustained low-grade ER stress activates not only the pro-inflammatory pathway [108] but also autophagy to compensate for the increased misfolded protein degradation exemplified by the increased *BECN1* gene expression. *BECN1* is a major autophagy activator gene whose increased expression is associated with the ER stress response [109,110,111,112] and its expression was also reduced by bromocriptine-QR therapy. Sustained low-grade cellular oxidative stress can lead to genomic damage that induces increased expression of *PIN1* to initiate DNA repair and a pro-inflammatory response in immunocytes [113]; *PIN1* expression was also reduced by bromocriptine-QR therapy. Activation of the ER UPR also induces the expression of antioxidant genes to assist with the refolding of unfolded/misfolded proteins and manage the increase in cellular ROS. Bromocriptine-QR treatment also reduced the mRNA expression of both the master antioxidant gene regulators, *OXR1*, *NRF2*, and *NQO1*, as well as multiple down-stream antioxidant target genes including *SOD1*, *CAT*, *GPX1*, *GPX4*, *GCH1* (that act collectively to combat cellular elevations in ROS), and *HMOX1* that are elevated in metabolic syndrome conditions [114,115,116,117,118,119,120,121,122,123,124,125,126]. *HMOX1* is an antioxidant that is induced by ROS and exerts protective effects against PBMC inflammation, macrophage foam cell formation, vascular inflammation, and dendritic cell-facilitated atherogenesis [123,124]. Collectively, the bromocriptine-QR-induced reductions in these antioxidant gene expressions indicate that bromocriptine-QR alleviates an elevated oxidative stress environment known to exist in cardiometabolic diseases such as T2D [76,77,80,81,82,83,84,85,86,89,101,102,105,106,107,127,128].

**Figure 7:**
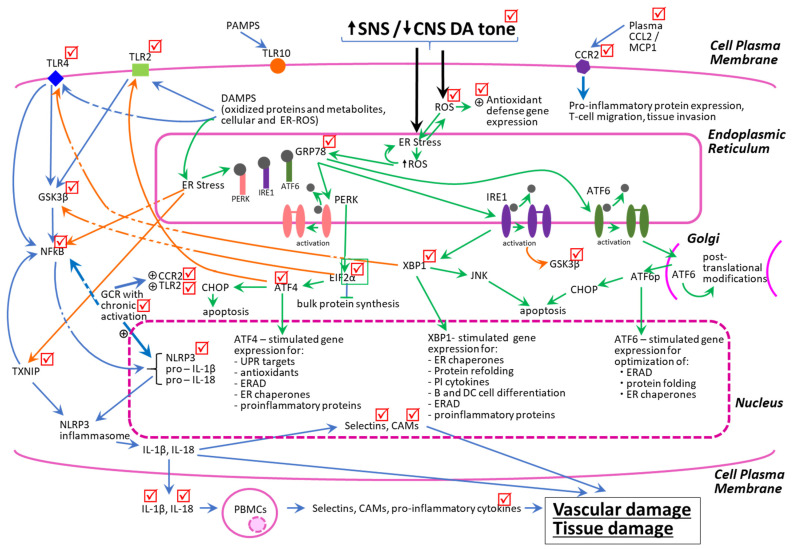
Schematic representation of the interacting dynamics of PBMC pro-oxidative and pro-inflammatory-state biochemical pathways. The check marks represent molecular sites of bromocriptine-QR action to reduce gene expression or plasma levels of pro-oxidative/pro-inflammatory factors. The green arrows represent ER stress-related actions, the blue arrows represent TLR-initiated pro-inflammatory pathways, and the orange arrows represent crosstalk activities between the ER stress and TLR-activated pathways leading to potentiation of vascular disease. All arrows indicate stimulation of gene expression and/or protein synthesis/activation of the target.

In addition to the reduction in chronic PBMC cellular and ER oxidative stress states, bromocriptine-QR also decreased both ER stress-associated and TLR-induced pro-inflammatory pathway proteins. As discussed above and in the Methods section, ER stress UPR proteins in PBMC can induce and activate the pro-inflammatory pathway proteins including *TLR2*, *TLR4*, *GSK3β*, *NFkB*, *TXNIP*, *NLPR3*, *BECN1*, *PIN1*, *CCR2*, and *GCR*. These same pro-inflammatory pathway proteins also are induced via TLR activation by DAMPs, which originate from the oxidation of cellular and extracellular matrix proteins. DAMPs stimulate the plasma membrane TLR 2 and 4 receptors that, via multiple parallel pathways including the activation of GSK3β, induce NFkB leading to increased expression of *TXNIP* and *NLRP3*, whose interaction allows the construction of the NLRP3 inflammasome that increases the production of several inflammatory cytokines (e.g., IL-1B and IL-18) and the secretion of extracellular adhesion molecules (L-selectin, vCAM, iCAM, and others) that stimulate monocyte trans-endothelial migration and promote tissue damage [37,43,82,108,127,129,130,131,132,133,134,135]. Importantly, the downstream molecular intermediates of the ER stress UPR that activate these pro-inflammatory proteins (e.g., *GSK3β*, *NFKB*, *TXNIP*, and *NLRP3*) also cross-activate these same pro-inflammatory targets within the TLR pathway (e.g., *ATF4* and *XBP-1*-mediated) [136,137] (see Figure 7).

Thus, under the conditions of chronic ROS stress to the PBMCs (resulting from elevated SNS tone, diminished CNS dopaminergic activity, the MS milieu, and diabetes), a positive reverberating feedback loop is created between the ER stress UPR and TLR inflammation pathway, which produces and maintains a low-grade systemic inflammatory state leading to vascular and tissue damage. Remarkably, bromocriptine-QR therapy simultaneously reduced the PBMC mRNA expression levels, not only of the above-mentioned ER stress proteins and oxidative stress response proteins, but also of the pro-inflammatory pathway proteins, *TLR2*, *TLR4*, *GSK3β*, *NFkB*, *TXNIP*, *NLPR3*, *BECN1*, and *PIN1*, and the adhesion molecules, *L-selectin* and *vCAM*, that these pathways induce. Bromocriptine-QR therapy did not, however, reduce *TLR10* expression, which is an orphan receptor that is activated by pathogen-associated molecular patterns (PAMPs) and assists in the innate immune response to infection. Lastly, the stimulation of the PBMC *CCR2* receptor by circulating/paracrine/autocrine CCL2 (MCP-1) is a potent stimulus for the PBMC pro-inflammatory activation and induction of adhesion molecule synthesis and secretion, whereas the nuclear GC receptor (*GCR*) is upregulated in response to the chronic pro-inflammatory state [129,130,131,132,133]. Moreover, in a chronic pro-inflammatory condition, *GCR* activation by corticosteroids potentiates further pro-inflammatory processes [129,130,131,132]. Bromocriptine-QR treatment reduced the mRNA expression of both of these signature pro-inflammatory receptors (*CCR2* and *GCR*), as well as the above-mentioned PBMC adhesion molecules (Figure 7).

Bromocriptine-QR induced widespread decreases in PBMC PO/PI gene expression, which were coupled with reductions in plasma levels of the pro-inflammatory chemokine MCP-1/CCL2, cytokines IL-1B and IL-18, and the pro-inflammatory hormone prolactin, elevations of which are associated with accelerated cardiometabolic disease [47,138,139,140,141,142,143,144,145,146,147,148,149,150]. Consistent with these treatment-induced reductions in PBMC and plasma pro-inflammatory genes and factors, respectively, bromocriptine-QR therapy also reduced the plasma CRP level by 45%, an indicator of low-grade systemic inflammation and a potential predictor of CVD, especially in T2D patients [151,152,153]. Also, bromocriptine-QR treatment reduced the plasma markers of the systemic oxidative stress- nitrotyrosine and TBARS. In metabolic syndrome, MCP-1, IL-18, and IL-1B are elevated and stimulate pro-inflammatory actions of tissue neutrophils and macrophages in vascular endothelial and smooth muscle cells. Bromocriptine-QR treatment reduced both the plasma MCP-1/CCL2 level and the expression of its PBMC receptor, *CCR2*, suggesting a dual inhibitory action on this inflammatory pathway. Although these plasma factors are influenced by several environmental conditions (such as diet, psychosocial stress, circadian phase—time of day, and others) that can cause within-group variation [154,155,156], bromocriptine-QR treatment nonetheless caused a significant reduction in their plasma levels.

**Figure 8:**
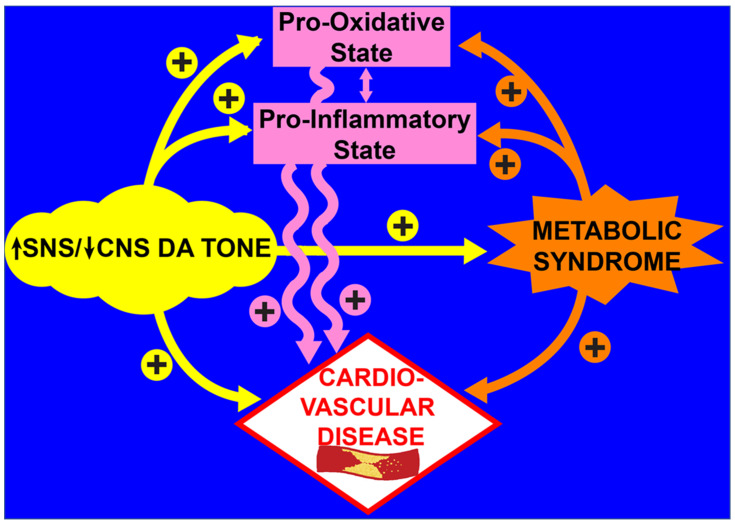
Schematic of interacting, parallel, and reinforcing pathways of increased SNS and decreased CNS dopaminergic tone in the development of cardiovascular disease.

As described above, numerous clinical and preclinical studies have documented that a PBMC and systemic pro-oxidative/pro-inflammatory condition typifies the T2D state and contributes to the cardiometabolic pathology of the disease [84,87,88,89,90,91,92,93,94,95,96,97,98,99,100,103,157,158,159,160,161,162,163,164]. The chronic metabolic syndrome milieu of T2D, including glucolipotoxicity, insulin resistance, and dyslipidemia, in turn, feeds back to potentiate the immune system’s PO/PI state, and, as the cycle churns, it precipitates damage to organs and the cardiovascular system (endothelial dysfunction, arteriosclerosis, atherosclerosis, fibrosis, myocardial infarction, heart failure) [14,37,38,40,41,42,44,46,47,48,49,76,77,78,79,80,81,82,83,84,85,86]. An external engine that can drive the rate of this cycle is an altered autonomic regulation typified by a simultaneous elevation of sympathetic tone and a CNS hypodopaminergic state. As discussed above, the increased SNS/decreased CNS dopamine activity stimulates the PO/PI state [19,20,21,22,23,24,25,26,27,28,29,30,31,32,33,34,35,36,50,51,52,53,54,55,56,57,58,59,60,61,62,63,64,65,66] and aggravates the MS milieu [1,165] increasing the PO/PI—MS—CVD cycle rate (see Figure 8).

It should be emphasized that these observed effects of elevated SNS/hypodopaminergic CNS activity can be manifested via alterations of the direct binding of norepinephrine and dopamine to the norepinephrine and dopamine receptors on PBMCs, as well as via their indirect CNS effects on the components of the (circadian) neuroendocrine axis. Circadian-timed treatment with the sympatholytic dopamine D2 receptor agonist, bromocriptine-QR, simultaneously decreased plasma norepinephrine and normetanephrine levels in association with declines in plasma pro-inflammatory factors, biomarkers of systemic oxidative stress, PBMC ER stress, the oxidative stress response, and inflammatory gene expression. This response to sympatholytic, dopamine agonist therapy in T2D subjects is not surprising given that previous studies have identified elevated sympathetic tone and decreased central nervous system dopaminergic activity as potent inducers of systemic immune inflammation at multiple tissue sites including the liver, kidney, vasculature, and heart [19,20,21,22,23,24,25,26,27,28,29,30,31,32,33,34,35,36,50,51,52,53,54,55,56,57,58,59,60,61,62,63,64,65,66]. In fact, the current study findings indicate a significant positive correlation between the bromocriptine-QR-induced reduction in plasma total measured catecholamines (norepinephrine plus normetanephrine) (in agreement with its sympatholytic activity) and its reduction in the PBMC gene expression of the ER stress-response gene, *EIF2α*, and also of the penultimate TLR and ER stress-responsive initiation factor for pro-inflammatory biochemical events- *NLRP3* in these study subjects. This effect was independent of weight or BMI changes, neither of which were altered by treatment in these study subjects [104].

Although the elevated sympathetic tone and decreased CNS dopaminergic activity are both drivers of hypertension, insulin resistance, postprandial hyperglycemia, and hypertriglyceridemia [1,165], which can contribute to increased CVD risk, available evidence indicates a substantial impact of the pro-oxidative/pro-inflammatory state in the development of CVD as described above. It follows, therefore, that the anti-inflammatory effect of bromocriptine-QR in T2D is likely to be a major contributor to the reduction in CV events observed with this therapy in T2D subjects [3,4,5,6]. Vascular inflammation contributes to endothelial dysfunction and is an established pathophysiologic abnormality that plays an important role in the development of CVD. In a separate study with this current subject group, bromocriptine-QR therapy did, in fact, improve endothelial dysfunction [104]. Additionally, immune inflammation is a significant contributor to heart failure, ischemic reperfusion injury, and peripartum cardiomyopathy, each of which has been shown to be improved by bromocriptine treatment [73,74,75,135,166,167,168,169,170,171,172,173,174].

Importantly, in preclinical studies of vascular disease, circadian-timed bromocriptine administration to reinstate the natural circadian peak in CNS dopaminergic activity at waking from daily sleep, which is reduced in insulin-resistant states (and causative in its genesis) [1,175], reduced aortic enzymes that potentiate the cellular generation of ROS and endothelial NOS uncoupling, two vascular pathophysiological events induced by a local pro-inflammatory environment and that contribute to endothelial dysfunction and CVD [11]. However, this bromocriptine effect was absent when administered outside of the natural CNS dopaminergic activity circadian peak window [11]. Preclinical studies have located a major aspect of the biological clock for the circadian rhythm of CNS dopaminergic activity to reside in dopaminergic neurons projecting from the supramammillary nucleus to the suprachiasmatic nucleus (SCN) [11,175,176,177,178,179,180,181]. The loss of the natural circadian peak of CNS dopaminergic activity at the SCN clock via direct neuronal neurotoxin micro-administration to the SCN area or high-fat feeding results in an elevated sympathetic tone that causes marked insulin resistance, glucose intolerance, and loss of hypothalamic glucose sensing, which is necessary for appropriate postprandial glucose disposal [178,180]. Further, reinstatement of this circadian peak of CNS dopaminergic activity in high-fat-fed animals by either appropriately circadian-timed direct dopamine administration to the SCN area or systemic bromocriptine administration restores normal glucose metabolism and sympathetic tone in these insulin-resistant animals but not when such administrations are made outside of the natural CNS dopaminergic circadian peak activity window [178,180]. Likewise, bromocriptine administration to obese insulin-resistant humans in the morning (time of the natural circadian peak of CNS dopaminergic activity at waking) improves postprandial insulin resistance but not so when it is administered in the evening [182]. Multiple studies have identified alterations in whole-body circadian rhythmicity associated with (and that may drive) CVD [183,184,185,186,187,188,189,190,191,192,193,194,195,196,197,198,199]. It may be that the “resetting” towards normal of CNS circadian dopaminergic aberrations that via the neuroendocrine axis resultantly reduces elevated SNS tone and improves metabolism [11,175,181,200,201,202] also resultantly resets towards normal those aberrant circadian immune activities that potentiate the PO/PI state. Consequently, both neurometabolic and neuroimmune responses to circadian timed dopaminergic stimulation may work in concert to drive/participate in the improvement in the immune PO/PI state and its cardiometabolic sequelae in T2D. This postulate is worthy of further investigation. In this regard, it should be noted that the present study’s effects of circadian-timed bromocriptine-QR therapy may be the consequence of an influence (via the circadian neuroendocrine axis) that directly reduced the PBMC PO/PI gene expression at the time of cell harvest (in the morning after an overnight fast) or an influence of a phase shift of the circadian rhythm of PBMC PO/PI gene expressions so that the trough (or otherwise lower) level in these daily gene expressions now occurred at this time of harvest (i.e., the treatment moved the PBMC PO/PI response rhythm out of phase with the neuroendocrine stimulus rhythms for inflammation to reduce the PO/PI gene expressions at the morning time of the cell harvest). It is also possible that, given the established circadian rhythm of PBMC gene expressions and activities [203,204,205,206], the anti-inflammatory bromocriptine-QR effect delineated herein underestimated its true effect as a consequence of circadian PBMC harvest time (i.e., harvest was not at the daily peak of the PO/PI gene expressions prior to treatment). Further studies are required and warranted for establishing the relationships between the circadian time of bromocriptine-QR administration and the drug’s influence on the circadian rhythms of PBMC PO/PI gene expressions and pro-inflammatory phenotypes.

The present study has several limitations including the relatively small sample size, absence of a placebo-treated T2D group, and lack of an NGT group with which to compare the baseline values. Moreover, the study population was entirely of Hispanic ethnicity, a group known to have increased rates of both T2D and vascular complications associated with T2D relative to the Caucasian population [207], and this may have affected the magnitude of the bromocriptine-QR response on the PO/PI state. However, it is well documented that T2D is a PO/PI state that potentiates CVD across ethnic populations. Further, even with the modest sample size, bromocriptine-QR caused significant reductions in a wide range of bona fide pro-oxidative/pro-inflammatory parameters and functions that were associated with a significant improvement in endothelial dysfunction previously reported in the same study subjects [104]. In as much as the present study findings are consistent with numerous previous studies reporting a pro-inflammatory role for elevated sympathetic tone and decreased CNS dopaminergic activity, the present results suggest that a larger, controlled study of the involvement of anti-oxidative/anti-inflammatory actions of circadian-timed bromocriptine-QR in improving the cardiometabolic status of T2D subjects across ethnic populations is warranted.

## 4. Materials and Methods

### 4.1. Study Design and Therapeutic Intervention Regimen

Description of the T2D study patients and study design of the therapeutic intervention with bromocriptine-QR has been previously described in detail [104]. Briefly, 15 Hispanic (11 females [8 of whom were post-menopausal at baseline]; 4 males) T2D subjects whose glycemia was poorly controlled (HbA1c > 7.5%) with a stable dose of liraglutide (1.2–1.8 mg/d; *n* = 15) plus metformin (*n* = 12) or low-dose glargine insulin (*n* = 3) comprised the study population. Subjects had a mean (±SEM) age = 57 ± 9 years, BMI = 33.4 ± 4.4 kg/m^2^, A1c = 8.3 ± 0.5%, and diabetes duration = 10.2 ± 5.6 years. Body weight (88 ± 13 kg) was stable (±3 lbs) for the 3 months prior to the study. Only subjects with a daytime feeding/night-time sleeping schedule were included. All subjects followed a standard ADA-recommended diet for diabetes, did not participate in any excessively heavy exercise programs, and were not taking any medications known to alter glucose metabolism (with the exception of metformin and insulin). Individuals with evidence of major organ system disease, diabetic proliferative retinopathy, and symptomatic neuropathy, as determined by physical examination, history, and screening laboratory tests, were excluded. Subjects were not taking any medications that would alter neurosynaptic function. Major exclusion criteria included individuals with evidence of major organ system disease, diabetic proliferative retinopathy, and symptomatic neuropathy. Subjects continued to take their normal pre-study dose of liraglutide plus either metformin or insulin throughout the study. Subjects were instructed to start Cycloset at a dose of 0.8 mg/day in the morning within two hours of waking. After 1 week, the daily Cycloset dose was increased by 0.8 mg in each subsequent week until the maximum dose of 3.2 mg once daily was reached after 4 weeks. In three patients, the final Cycloset dose was decreased to 2.4 mg/day for the symptoms of orthostatic hypotension, lightheadedness, nausea, and vomiting. Baseline blood samples for plasma analyses and PBMC isolation/analyses were taken in the morning after an overnight fast before and 4 months after initiation of bromocriptine-QR therapy. The 4-month sample size was obtained in the morning before that day’s dose of bromocriptine-QR was ingested. The study was approved by the University of Texas Health Science Center at San Antonio (UTHSCSA) IRB and informed written consent was obtained from all participants prior to the study. All clinical study procedures were conducted at the Clinical Research Center at the Texas Diabetes Institute (TDI), University Hospital System.

### 4.2. Rationale for PBMC Analyses of Specific Gene mRNA Expression Levels as Assessment of the PO/PI State in T2D

The ER is a complex organelle that is involved in protein biogenesis, appropriate tertiary structure acquisition (folding), and trafficking within the cell (proteostasis). ER proteins and small molecules function in concert, in large part via oxidation-reduction reactions, to maintain an ER environment that is favorable for the execution of its proteostasis functions. However, pathophysiological stresses present in individuals with MS (including hyperglycemia, elevated plasma FFA levels, oxidized LDL, triglycerides, and other biomolecules), as well as increased intracellular reactive oxygen species (ROS) from chronically elevated SNS stimulation, mitochondrial dysfunction, and an overactive ER, disrupt the ER machinery and cause ER stress. This ER stress is characterized by the accumulation of unfolded and misfolded proteins within the ER that trigger the canonical unfolded protein response (UPR) [89,102,105,106,107]. The UPR functions to slow protein synthesis, increase unfolded/misfolded protein degradation, and correct misfolded protein structure. However, if the UPR cannot keep pace with the level of protein misfolding, an ER redox imbalance-driven process ensues and stimulates the expression of pro-inflammatory genes, accelerates autophagy, and causes cellular apoptosis. Since the UPR itself is ROS-generating, as it escalates in the presence of the metabolic syndrome milieu or direct SNS noradrenergic stimulation, a vicious self-sustaining cycle of ER stress and pro-inflammatory protein secretion is manifested [89,102,105,106,107]. The ER stress within PBMCs contributes to their PO/PI phenotype that potentiates local and systemic vascular disease and CVD [166,167,208,209,210,211,212,213] and insulin resistance [76,102,214,215,216].

Overactivation of certain cellular toll-like receptors (TLRs) can potentiate the PBMC PO/PI phenotype. TLRs are a family (TLRs 1–10) of the plasma membrane and endosomal pattern recognition receptors that on immune cells activate several arms of the innate immune defense response against pathogens [217,218]. TLR overactivation by PAMPs, such as bacterial or viral particles [217,218,219,220], or by endogenous damage/danger-associated molecular pattern (DAMP) molecules (heat-shock proteins, fatty acids, oxidized phospholipids, oxidized LDL, extracellular matrix breakdown products, nucleic acid fragments, intracellular oxidized molecules, ROS) [80,221,222] activate the TLR signaling cascade to express and activate downstream potent pro-inflammatory effectors including *glycogen synthase kinase 3 beta (GSK3β)*, *nuclear factor kappa-B p65 subunit (NFkB), thioredoxin interacting protein (TXNIP)*, *and the NLR family pyrin domain-containing protein 3 (NLRP3)* inflammasome (collectively termed sterile inflammation). Chronic activation of these TLR response pathways results in inflammatory cytokine and adhesion molecule production and secretion to potentiate vascular and tissue inflammation, promoting CVD [80,217,218,219,220,221,222,223,224]. Such chronic DAMP stimulations of PBMC *TLR 2* and *4* can also potentiate the metabolic syndrome itself [80,221,222]. Importantly, there is extensive crosstalk between the ER stress-response and TLR activation pathways such that the transcription factors and enzymes of each of these pro-inflammatory pathways can cross over and stimulate the other pro-inflammatory pathways at various points to amplify the overall inflammatory response to accelerate cardiovascular disease [136,137].

The PBMC analyses in the present study [104] were performed on quickly frozen (in liquid nitrogen) cell samples. In vivo, the activation state of the enzymes and redox fluxes of biomolecules in their cellular micro-environments are rapidly changing and difficult to quantitate [225,226] and such measurements from harvested, processed, and frozen tissues are inappropriate. The PBMC transcriptome reflects the system-wide inflammatory state, including the effect of changes in response to pharmaceutical intervention [227,228,229]. Consequently, a realistic evaluation of the sustained changes in the redox status in frozen cell samples is best obtained from the measures of the gene expression levels for long-lived proteins, which participate in or are responsive to a sustained PO/PI cellular status, including ER stress proteins and ROS-responsive anti-oxidative proteins, which, in composite, can provide an overview of the impact of drug interventions toward or away from a chronic pro-oxidative/pro-inflammatory pathophysiological condition [77,80,84,85,164,230,231,232,233]. Cellular inflammatory responses to **chronic** oxidative stress that potentiate CVD are long-lived and can be assessed by measures of PBMC pro-inflammatory protein gene expression [76,77,78,80,82,101,136,137,222]. Several critical elements of this cellular PO/PI gene response system have been delineated and the present study evaluates the PBMC expression level of select mRNAs for several proteins established to be critically involved in intersecting the pathways of the cellular redox balance and inflammatory status. These proteins include several ER stress-response proteins (*GRP78*, *EIF2α*, *ATF4*, *XBP1*, *ATF6*, *PERK*, *CHOP*, *BECN1*, *PIN1*), master regulators of oxidative stress-response proteins (*NRF2*, *OXR1*, *NQO1*), downstream key oxidative stress-response [antioxidant] enzymes (*SOD1*, *SOD2*, *GSR*, *NOS2*, *CAT*, *GPX1*, *GPX4*, *GCH1*, *HMOX1*), protein activators of the TLR inflammatory pathway (cell membrane and nuclear receptor activators of inflammation, master pro-inflammasome transcription factors, and inflammatory cytokine transcription factors including *TLR2*, *TLR4*, *TLR10*, *GSK3β*, *NFkB*, *MAPK8/JNK*, *TXNIP*, *NLRP3*, *CCR2*, *GCR*), and pro-inflammatory cellular/tissue adhesion molecules (*SELL*, *VCAM1*, *ICAM1*) to derive a composite fingerprint of the cellular redox/inflammatory state before and after 4 months of bromocriptine-QR therapy. Under the conditions of chronic low-grade inflammation from a systemic oxidative stress environment, PBMCs exhibit ER stress, which is exemplified by an increase in the stress sensor, chaperone protein GRP78, that upon increased cellular redox stress releases the three bound protein activators of the ER stress response—*PERK*, IRE1, and ATF6. *PERK* phosphorylates EIF2α, which in turn (i) induces ER actions to handle the increased workload by slowing bulk protein synthesis and (ii) activates ATF4 to initiate ER-associated misfolded protein degradation activity, autophagy, antioxidant gene expression, and UPR gene expression all to potentiate cell survival. The IRE-1 protein functions via the induction and activation of *XBP1* to upregulate mRNAs of the UPR, stimulate autophagy, and activate the inflammatory response to ER stress. The ATF6 protein is functionally associated with improved protein folding, post-translational modifications, and translocation to and from Golgi, but also activates *XBP1* resulting in pro-inflammatory gene expression [89,105,106,107,108,230,234,235]. ER stress elements, including EIF2α, ATF4, and XBP1 (and TLR activation cascade pathways) can induce and activate *GSK3β*, which acts as a positive regulator of the inflammatory process by inducing multiple transcription factors *(e.g., mitogen-activated protein kinase 14*, *p38*, *JNK1/2*, *c-Jun*, *ATF-2*, *and/or NFkB*) and increasing the production of pro-inflammatory cytokines including IL-1B and IL-18 [105,136,137,236,237]. In response to sustained ER stress, *beclin 1 (BCN1)* protein is overexpressed and augments autophagy to alleviate the buildup of misfolded proteins [109,110,111,112] in order to potentiate cell survival. Also, upon sustained ER stress in immunocytes, *PIN1*, *a proline-associated isomerase* whose over-expression is associated with a range of diseases, is upregulated and promotes inflammatory cytokine expression and apoptosis (reviewed in ref [113]). Although increased local and intracellular ROS can initiate cellular ER stress, the ER stress response also generates ROS. Thus, under the conditions of sustained low-grade local ROS increase, PBMCs respond by upregulating antioxidant pathways [89,102,105,106,107]. *NRF2* (including its immediate target *NQO1*) and *OXR1* act as master transcription factors for the downstream induction of multiple antioxidant genes in response to ROS increases and ER stress [114,115,116,117,118,119]. The downstream ROS-response antioxidant gene whose expression is increased are (i) *SOD1* and *2* as well as *CAT* that decrease ROS and the long-lived pro-oxidant H_2_O_2_, respectively, and (ii) the glutathione-dependent-reducing enzymes *GSR*, *GPX1*, and *GPX4* that reduce elevated levels of H_2_O_2_, ROS, and lipid peroxidation levels and thus combat ROS-induced inflammation and apoptosis [120,121,122]. *HMOX1* is another potent antioxidant enzyme that is induced by ER stress and catalyzes the metabolism of plasma-free heme, a potent oxidizing agent that induces ER stress, to generate cytoprotective biliverdin, carbon monoxide, and ferrous ion. *HMOX1* is upregulated by *NRF2* in response to cellular (ER) oxidative stress and has potent anti-inflammatory and anti-apoptotic activities (reviewed in ref [123,124]). *GCH1* functions as the rate-limiting enzyme in tetrahydrobiopterin synthesis and its expression is induced in response to the redox stress of elevated H_2_O_2_ to generate tetrahydrobiopterin, a cofactor for *iNOS (NOS2)* (and *endothelial NOS*) and subsequent NO production to inhibit an existing inflammatory condition [125,126].

The cellular PBMC sterile pro-inflammatory state (inflammation not induced by microbes) is driven largely via TLRs’ activation by DAMPs (damaged intracellular proteins, phospholipids, and extracellular matrix components), which are generated by local and intracellular oxidative stress reactions, including those emanating from the ER. TLRs can also be activated by dysregulated metabolites such as saturated fatty acids, oxidized LDL, oxidized phospholipids, homocysteine, and glucose [80,217,218,220,221,222]. Chronic activation of *TLR2* and/or *TLR4* by DAMPs initiates a cascade of parallel signal transduction reactions leading to the induction and activation of *GSK3β* and its downstream targets *NFkB* and *NLRP3*, which associate with *TXNIP* (induced by ER stress and increased cellular ROS) to form the NLRP3 inflammasome enzyme complex that generates pro-inflammatory cytokines including IL-1β and IL-18 [83,238,239,240,241,242,243,244]. Chronic elevation of these two cytokines stimulates the synthesis of the vascular immunocyte adhesion molecules (e.g., L-selectin, vCAM, iCAM), which potentiate immunocyte tissue invasion and migration [242,245,246,247,248,249]. The cell surface receptor CCR2, when activated by binding to its circulating ligand, CCL2/MCP-1, initiates PBMC pro-inflammatory cytokine production, migration, and tissue invasion, which leads to vascular and organ inflammatory damage (e.g., atherosclerosis, fibrosis, apoptosis, necrosis) [138,144,250,251,252,253,254]. The nuclear receptor *GCR*, when stimulated in response to acute stress, acts as a potent anti-inflammatory signal transducer to reduce inflammatory cytokine production. However, if overactivated by its ligand corticosteroid hormones, GCR can increase *CCR2*, *TLR2*, and *NLRP3* expression to support a pro-inflammatory phenotype under *chronic* stress conditions such as MS and T2D [129,130,131,132,133]. In total, such vascular inflammation activation from systemic/PBMC pro-oxidative stress augments vascular endothelial dysfunction, smooth muscle cell proliferation, arteriosclerosis, and cardiomyocyte damage/death [37,43,81,82,86,101,134,135,144,166,167,208,209,210,211,212,213,214,215,216,241,242,250,251,252,253,254]. Most importantly, the members of the UPR pathways of ER stress can activate the TLR pro-inflammatory cascade and vice-versa creating a self-sustaining pro-oxidative/pro-inflammatory condition, ultimately producing organ damage [136,137]. In insulin-resistant states such as T2D, the above PBMC ER stress, ROS-responsive antioxidant and pro-inflammatory pathways, and operative protein levels are elevated [37,38,39,40,41,76,77,78,79,80,81,84,85,98,102,103,115,134,144,157,158,159,160,161,162,163,164,214,215,216,222,230,231,239,241]. Figure 7 presents a schematic representation of the interacting dynamics of these PBMC pro-oxidative and pro-inflammatory pathways and activities and the impact of bromocriptine-QR thereupon.

### 4.3. Biochemical Assays of Blood Samples

Blood samples were collected in EDTA-preconditioned tubes for the isolation of plasma, which was used for analyses of all humoral analytes.

#### 4.3.1. Assessment of SNS Tone Markers (Norepinephrine, Normetanephrine)

Plasma norepinephrine was determined with an ELISA kit (Cat# 17-NORHU-E01-RES, ALPCO, Salem, NH), and plasma normetanephrine was determined with an ELISA kit (Cat# NMN31-K02 Eagle Bio, Nashua, NH).

#### 4.3.2. Assessment of Plasma Oxidative Stress and Pro-Inflammatory Markers

Plasma Thiobarbituric Acid Reactive Substances (TBARS) were determined with a TBARS assay kit (Cat# 700870, Cayman Chemicals, Ann Arbor, MI, USA). Plasma nitrotyrosine was determined with a nitrotyrosine assay kit (Cat# STA-305, Cell Biolabs, San Diego, CA, USA). Plasma IL-1b was determined with a ProQuantum immunoassay (Cat# A35574, ThermoFisher, Waltham, MA, USA). Plasma IL-6 and IL-18 were determined with a Legendplex immunoassay (Cat# 740118, BioLegend, San Diego, CA, USA). Plasma prolactin was determined with an ELISA kit (Cat# DPRL00, R&D Systems, Minneapolis, MN, USA). Plasma CRP level was assayed with an ELISA kit (Cat # DCRP00, R&D Systems, Minneapolis, MN, USA).

### 4.4. Assessment of PBMC Pro-Oxidative/Pro-Inflammatory Status

#### 4.4.1. PBMC Isolation

Blood samples were collected from study subjects after an overnight fast at baseline and after 4 months of bromocriptine-QR therapy as described above. PBMN cells were isolated from 5 mL of EDTA-treated blood within two hours with Polymorphprep (Axis-Shield, Oslo, Norway) solution containing sodium diatrizoate 13.8% (*w/v*) and Dextran 500 8.0% (*w/v*) by centrifuging the samples layered over Polymorphprep at 450–500× *g* for 30–35 min in a swing-out rotor at 18–22 °C per the manufacturers’ instructions. PBMN cells were collected, washed in phosphate-buffered saline (PBS), and stored in liquid nitrogen at −196 °C before analysis.

#### 4.4.2. PBMC Gene Expression Analysis by Real-Time qPCR—mRNA Extraction and PCR Methods including Primers

Total RNA was isolated from PBMN cells with Trizol Reagent (ThermoFisher, Waltham, MA, USA Cat# 15596026). One mL of Trizol Reagent was added to frozen PBMN cells, cells were homogenized, and total RNA was isolated per the manufacturer’s instructions and resuspended in RNAse-free water. Total RNA quantity and purity were determined by UV spectroscopy, and then genomic DNA was digested with a double-strand specific thermolabile DNase, and cDNA was synthesized using SuperScript IV VILO MasterMix with ezDNAse (ThermoFisher, Waltham, MA, USA Cat# 11766050) using 2 ug of total RNA per sample in a 20 μL reaction. Real-time qPCR was performed with a Taqman fast advance master mix (ThermoFisher, Waltham, MA, USA Cat# 4444964) on an AriaMX (Agilent, Santa Clara, CA, USA) qPCR instrument with the following primer/probe sets (ThermoFisher, Waltham, MA, USA):

##### ER Stress-Associated Unfolded Protein Response Genes

*ATF4 (Activating Transcription Factor 4)* Cat# Hs00909569_g1

*ATF6 (Activating Transcription Factor 6)* Cat# Hs00232586_m1

*BECN1 (Beclin 1)* Cat# Hs01007018_m1

*BiP/GRP78 (Heat-Shock Protein Family A (Hsp70) Member 5)* Cat# Hs99999174_m1

*CHOP (DNA Damage Inducible Transcript 3)* Cat# Hs01090850_m1

*EIF2α (Eukaryotic Translation Initiation Factor 2, Subunit 1 Alpha)* Cat# Hs00187953_m1

*GSK3β (Glycogen Synthase Kinase 3 Beta)* Cat# Hs01047719_m1

*NFkB (NF-kappa-B p65 subunit)* Cat# Hs01042014_m1

*PERK (Eukaryotic Translation Initiation Factor 2 Alpha Kinase 3)* Cat# Hs00984003_m1

*PIN1 (Peptidylprolyl Cis/Trans Isomerase, NIMA-Interacting 1)* Cat# Hs01598309_m1

*TXNIP (Thioredoxin Interacting Protein)* Cat# Hs01006897_g1

*XBP1 (X-Box Binding Protein 1 functional variant)* Cat# Hs03929085_g1

##### Oxidative Stress Response (Antioxidant) Genes

*CAT (Catalase)* Cat# Hs00156308_m1

*GCH1 (GTP Cyclohydrolase 1)* Cat# Hs00609198_m1

*OXR1 (Oxidation resistance gene 1)* Cat# Hs00757847_g1

*GPX1 (Glutathione peroxidase 1)* Cat# Hs01028922_g1

*GPX4 (Glutathione peroxidase 4)* Cat# Hs00157812

*GSR (Glutathione–Disulfide Reductase)* Cat# Hs00167317

*HMOX1 (Heme Oxygenase 1)* Cat# Hs01110250_m1

*NQO1 (NAD(P)H Quinone Dehydrogenase 1)* Cat# Hs01045993_g1

*NRF2 (Nuclear factor erythroid 2-related factor 2)* Cat# Hs00975961_g1

*SOD1 (Superoxide dismutase 1, cytoplasmic)* Cat# Hs00533490_m1

*SOD2 (Superoxide dismutase 2, mitochondrial)* Cat# Hs00167309_m1

##### Pro-Inflammatory Genes

*CCR2 (C-C Motif Chemokine Receptor 2)* Cat# Hs00356601_m1

*GCR (Glucocorticoid receptor)* Cat# Hs00353740_m1

*GSK3β (Glycogen Synthase Kinase 3 Beta)* Cat# Hs01047719_m1

*MAPK8/JNK (Mitogen-Activated Protein Kinase 8)* Cat# Hs01548508_m1

*NFkB (NF-kappa-B p65 subunit)* Cat# Hs01042014_m1

*NLRP3 (NLR Family Pyrin Domain-Containing Protein 3)* Cat# Hs00918082_m1

*TLR2 (Toll-like receptor 2)* Cat# Hs00610101_m1

*TLR4 (Toll-like receptor 4)* Cat# Hs01060206_m1

*TLR10 (Toll-like Receptor 10)* Cat# Hs01675179_m1

*TXNIP (Thioredoxin Interacting Protein)* Cat# Hs01006897_g1

##### Secretory Adhesion Genes

*ICAM1 (Intercellular Adhesion Molecule 1)* Cat# Hs00164932_m1

*SELL (L-selectin)* Cat# Hs00174151_m1

*VCAM1 (Vascular Cell Adhesion Molecule 1)* Cat# Hs01003372_m1

##### Housekeeping Gene

*RPS17 (Ribosomal Protein S17)* Cat# Hs00734303_g1

Cq values were calculated with Agilent Aria software version 1.5. The relative expression of genes was calculated using the 2^−ΔΔCq^ method as previously described [255]. *Ribosomal Protein S17 (RPS17)* has been shown to be a good reference gene for normalizing gene expression in PBMCs [256] and was used as the internal reference. Relative gene expression was calculated as a fold-change from the control samples.

### 4.5. Statistical Analyses

Statistical differences in the changes from the baseline of the PBMN gene expression and plasma biochemical parameters were first analyzed for normality with the Shapiro–Wilk test and then evaluated using a Student’s 2-tailed paired *t*-test for normally distributed data or Wilcoxon signed rank test for non-normal data unless noted otherwise in the Results section. A *p* value < 0.05 was accepted as statistically significant; results that were not statistically significant are labeled as not significant (NS). In certain instances, wherein a downstream target of a significantly affected gene or biochemical parameter was examined, a Student’s 1-tailed test was additionally employed to test for the significance of the same directional change. All data are expressed as the mean ± SEM of the study population. Statistical analysis was performed with SigmaPlot Version 14.5 (Systat, San Jose, CA, USA).

Correlation analyses were performed to evaluate the relationship between the change from baseline to week 24 of bromocriptine-QR treatment in the plasma total measured catecholamines (norepinephrine plus normetanephrine) level with such treatment changes in the expression of select PBMC genes representing key focal activation points for (a) the effector response to endoplasmic reticulum stress—(*EIF2α*), (b) the oxidative stress defense (master antioxidant gene transcription factor—*NRF2*), and (c) the TLR and ER stress-responsive penultimate initiation factor required for pro-inflammatory biochemical events (*NLRP3*). Also, the relationship between the changes from baseline to week 24 of bromocriptine-QR treatment in the fasting reactive hyperemia index (RHI) with such treatment changes in HbA1c was evaluated. Pearson’s correlation analyses were employed for normally distributed variables and non-parametric Kendal’s Tau-b analyses were employed for non-normally distributed data (change in HbA1c).

## 5. Conclusions

In conclusion, the present study findings of a circadian-timed bromocriptine-QR simultaneous reduction in sympathetic tone and an immune and systemic pro-inflammatory/pro-oxidative state provide potential biological mechanisms that can contribute to the cardioprotective effects of bromocriptine-QR therapy observed in T2D subjects [3,4,5,6].

## Figures and Tables

**Table 1:** Baseline characteristics.

Parameter	Mean ± SEM
Age	57 ± 9 years
Baseline HbA1c	8.3 ± 0.3%, all subjects had baseline HbA1c >7.5%
Duration of diabetes	10.2 ± 5.6 years
Body weight	88 ± 13 kg
BMI	33.4 ± 4.4 kg/m^2^,
Fasting plasma glucose	145 ± 3 mg/dL
Fasting plasma insulin	20.0 ± 2.1 μU/mL
Fasting plasma C-peptide	4.8 ± 0.3 ng/mL
Fasting FFA	530 ± 12 μmol/L
Heart rate	74 ± 8 bpm
Systolic blood pressure	134 ± 4 mm Hg
Diastolic blood pressure	78 ± 3 mm Hg
Mean arterial blood pressure	97 ± 5 mm Hg
Sex	11 females (8 post-menopausal), 4 males
Concomitant diabetes medications	
Subjects on liraglutide	1.2–1.8 mg/day; *n* = 15
Subjects on metformin	*n* = 12
Subjects on insulin glargine, low dose	*n* = 3

## Data Availability

The data presented in this study will be made available on request to the corresponding author.

## Abbreviations

Abbreviations used in Figure 7	
CNS	Central nervous system
DA	Dopaminergic
DAMP	Damage-associated molecular pattern
ER	Endoplasmic reticulum
ERAD	ER-associated degradation
PBMC	Peripheral blood mononuclear cells
PI	Pro-inflammatory
ROS	Reactive oxygen species
SNS	Sympathetic nervous system
UPR	Unfolded protein response
Genes mentioned in Figure 7	
*ATF4*	*Activating Transcription Factor 4*
*ATF6*	*Activating Transcription Factor 6*
*BiP/GRP78*	*Heat-Shock Protein Family A (Hsp70) Member 5*
*CCR2*	*C-C Motif Chemokine Receptor 2*
*CHOP*	*DNA Damage Inducible Transcript 3*
*EIF2α*	*Eukaryotic Translation Initiation Factor 2, Subunit 1 Alpha*
*GCR*	*Glucocorticoid receptor*
*GSK3β*	*Glycogen Synthase Kinase 3 Beta*
*ICAM1*	*Intercellular Adhesion Molecule 1*
*IRE1*	*Endoplasmic Reticulum to Nucleus Signaling 1*
*MAPK8/JNK*	*Mitogen-Activated Protein Kinase 8*
*MCP1/CCL2*	*Monocyte Chemoattractant Protein 1*
*NFkB*	*NF-kappa-B p65 subunit*
*NLRP3*	*NLR Family Pyrin Domain-Containing Protein 3*
*PERK*	*Eukaryotic Translation Initiation Factor 2 Alpha Kinase 3*
*TLR2*	*Toll-like receptor 2*
*TLR4*	*Toll-like receptor 4*
*TLR10*	*Toll-like Receptor 10*
*TXNIP*	*Thioredoxin Interacting Protein*
*VCAM1*	*Vascular Cell Adhesion Molecule 1*
*XBP1*	*X-Box Binding Protein 1 functional variant*
